# No Evidence of Off-label Use of Olodaterol and Indacaterol in Denmark, France, and the Netherlands: A Drug Utilization Study

**DOI:** 10.1038/s41598-019-57397-5

**Published:** 2020-01-17

**Authors:** Cristina Rebordosa, Eline Houben, Kristina Laugesen, Ulrich Bothner, Jukka Montonen, Jaume Aguado, Jetty A. Overbeek, Vera Ehrenstein, Joelle Asmar, Laura Wallace, Alicia W. Gilsenan

**Affiliations:** 1 RTI Health Solutions, Av. Diagonal 605, 9-1, 08028 Barcelona, Spain; 2 Research Triangle Park, NC North Carolina, USA; 3 PHARMO Institute for Drug Outcomes Research, Van Deventerlaan 30-40 3528 AE, Utrecht, Netherlands; 4 Department of Clinical Epidemiology, Aarhus University Hospital, Olof Palmes Allé 43-45, 8200 Aarhus N, Denmark; 5 IQVIA, RWI, Tour D2, 17 Bis Place des Reflets, TSA 64567, 92099 La Défense Cedex, France; 6 Boehringer Ingelheim International GmbH, Binger Str. 173, 55216 Ingelheim am Rhein, Germany; 7 Boehringer Ingelheim International GmbH, 900 Ridgebury Road, Ridgefield, CT 06877 USA

**Keywords:** Drug regulation, Asthma, Epidemiology

## Abstract

To characterize the use of olodaterol and indacaterol in clinical practice and to quantify the off-label use in asthma. Drug utilization study of new users of olodaterol or indacaterol between 2014 and 2017 in the PHARMO Database Network in the Netherlands, the Danish population registers, and the IMS Real-World Evidence Longitudinal Patient Database panels in France. On-label use was defined as use among adults with a recorded diagnosis of COPD. Off-label use was defined as use among adults with a recorded diagnosis of asthma without a recorded diagnosis of COPD or as use among patients aged ≤18 years. Potential off-label use was defined as no recorded diagnosis of either COPD or asthma. The study included 4,158 new users of olodaterol and 9,966 new users of indacaterol. Prevalence of off-label use ranged from 3.5% for both drugs to 12.4% for olodaterol and 11.9% for indacaterol. Prevalence of on-label use ranged from 47.8% to 77.7% for olodaterol and from 28.7% to 70.1% for indacaterol. The remaining new users of olodaterol and indacaterol were classified as potential off-label users, with prevalence ranging from 17.3% to 48.6% for olodaterol and from 20.5% to 66.6% for indacaterol. This study provides no evidence of a major concern in Europe for olodaterol or indacaterol for off-label use in asthma or for pediatric use.

## Introduction

Chronic obstructive pulmonary disease (COPD) affects 174 million people worldwide^1^. It is a characterized by persistent airflow limitation that is due to airway and/or alveolar abnormalities usually caused by significant exposure to noxious particles or gases including smoking^2^. Four inhaled long-acting beta2-agonists (LABAs) (salmeterol, formoterol, indacaterol, and olodaterol) are approved for long-term maintenance treatment in patients with COPD. Salmeterol and formoterol are also approved for use in asthma. Olodaterol was first approved in the countries of the European Union since 2013 and is indicated as a maintenance bronchodilator treatment to relieve symptoms in adult patients with COPD^3^. The use of LABAs as monotherapy in asthma without COPD, without a primary anti-inflammatory controller medication, e.g., an inhaled glucocorticosteroid (ICS), has been associated with increased morbidity and mortality^4,5^. There were no such concerns if the patient has COPD and concomitant asthma, an overlap of asthma and COPD, or COPD alone. Therefore, the health authorities of the European Union (EU)/European Economic Area Member States requested the conduct of a post-approval drug utilization study to characterize the use of olodaterol in clinical practice and assess the potential off-label use of olodaterol in asthma (see study protocol at EU PAS Register # EUPAS 17386)^6^. The main objectives of the study were to describe the baseline characteristics of patients initiating on olodaterol and to quantify off-label use of olodaterol to treat asthma in three countries in Europe. To provide a meanin gful context for the results, we also describe characteristics and off-label use of indacaterol, the other LABA not indicated to treat asthma.

## Methods

### Study design

We conducted a multinational, cross-sectional drug utilization study using information routinely collected in health care databases of new users of olodaterol or indacaterol. The study was conducted using health care databases in three countries: the PHARMO Database Network in the Netherlands (PHARMO overall and PHARMO General Practitioner [GP]) (www.pharmo.com)^7^, the Danish population registers in Denmark^8–11^, and the IMS Health Information Solutions Real-World Evidence Longi tudinal Patient Database (IMS RWE LPD) (GP panel and pulmonologist panel) in France^12^. Characteristics of the health care databases are described in Supplemental Table 1. Briefly, PHARMO overall includes electronic medical records (EMRs), of hospitalizations and outpatient pharmacy dispensings for more than 4 million residents of a well-defined population in the Netherlands (approximately 25% of that country’s population) for an average of 10 years. The PHARMO-GP is a 25% subset of the PHARMO overall that also includes EMRs from GPs. The Danish population registers used in this study were the Danish National Patient Register^9^, an administrative registry tracking hospitalizations and outpatient hospital visits, and the Danish National Health Services Prescription Database, which includes data on dispensings from outpatient pharmacies^10^. The IMS RWE LPD panels include EMRs from routine clinical practice from a subset of GPs (GP panel) and pulmonologist (Pulmonologist panel) in France. The study period started on the date of olodaterol launch in each country and ended on the latest date the data were available at the time of each final data extraction. The study periods included in the final data extraction for each data source were from March 1, 2014, to December 31, 2016, in PHARMO and the Danish population registers and from October 1, 2015, to November 30, 2017, in the IMS RWE LPD panels. The time periods vary by data source due to time lag in availability of data to researchers. The study was carried out in accordance with relevant guidelines and regulations. The study protocol was approved by the Institutional Review Board (IRB) and by the scientific and ethic committees required by each database. The need for informed consent was waived by the IRB.

### Study population

Patients were included in the study if they fulfilled all the following criteria: (1) received a first prescription/dispensing for single-agent formulations of olodaterol or indacaterol during the study period (new users, with no prescriptions/dispensings ever before) and (2) had at least 12 months of continuous enrolment in the study health care databases preceding the index date, i.e., the date of the first prescription/dispensing for olodaterol or indacaterol. Because the study aimed to assess the use of olodaterol and indacaterol in regular clinical practice, no exclusions regarding age, sex, or comorbidity were defined. However, individuals with missing or implausible (e.g., age over 120 years) values for age or sex were excluded.

### On-label, off-label, and potential off-label definitions

Patients were classified in three mutually exclusive groups according to their indication. *On-label users* were defined as patients aged 18 years or older with a recorded diagnosis of chronic bronchitis, emphysema, or “other COPD” (a recorded diagnosis of COPD without specifying chronic bronchitis or emphysema) at any time before the index date or up to 30 days after the index date. A time window of 30 days after index date was used to allow for a diagnosis to be recorded in the data sources when prescriptions/dispensings may be done by another physician^13^. Because COPD can occur in association with asthma, patients aged 18 years or older with a recorded diagnosis for both COPD and asthma were also considered on-label^14–16^. *Off-label users* were defined as patients aged 17 years or younger or patients aged 18 years or older with a recorded diagnosis of asthma in the absence of a recorded diagnosis for COPD at any time before or up to 30 days after the index date (see codes used to define COPD and asthma in Supplemental Table 2). *Potential off-label users* were defined as the remaining patients aged 18 years or older with no diagnosis of COPD and no diagnosis of asthma recorded at any time before or up to 30 days after the index date. An alternative definition of COPD (i.e., probable COPD) was applied to identify patients who likely had true COPD but for whom no recorded diagnosis was found in the available data source. Probable COPD was defined as the subset of potential off-label users that had at least two prescriptions/dispensings for a LABA, long-acting muscarinic antagonist (LAMA), or ICS (or combinations) after the age of 40 years but not before^17,18^. Review of a sample of 100 patient profiles, i.e. EMRs in chronological order, per drug in each data source was performed to confirm the appropriate performance of the algorithm to identify COPD and asthma, that no codes had been missed, and that the appropriate time windows were being used.

### Patient characteristics

New users of olodaterol and new users of indacaterol were characterized at the index date according to demographic variables (age and sex), lifestyle characteristics as available in each data source (Supplemental Table 3) (smoking, obesity, and alcohol consumption), respiratory and nonrespiratory comorbidities, comedications, and COPD severity. Covariates were ascertained based on all information available before the index date, except for comedications and COPD severity that were ascertained up to one year before index date.

Severity of COPD was evaluated among new users of olodaterol or of indacaterol who had a recorded diagnosis of COPD and were aged 40 years or older before the index date. Severity of COPD was evaluated only in the Netherlands and in Denmark and was not evaluable in France due to lack of information on hospitalization. In this study, severity of COPD was defined using a modified version of the algorithm developed by Verhamme, *et al*.^19^ and considering the updated recommendations by the Global Initiative for Chronic Obstructive Lung Disease (GOLD)^2^. Severity of COPD was determined based on information on intermittent versus regular bronchodilator medications use, exacerbations with and without hospitalizations, presence of emphysema, and use of nebulizer and oxygen therapy in the last 12 months prior to the index date (see criteria in Supplemental Table 4).

### Statistical analysis

The main analysis estimated the prevalence of off-label use among new users of olodaterol and indacaterol during the overall study period in each data source. Data describing the medical history and comedications of the study population are presented as counts, percentages, medians, and interquartile ranges, as appropriate. All data sources followed a common protocol and analysis plan. Data extraction, cohort selection, variable manipulation, and analysis were performed using SAS version 9.2 or higher.

### Ethical approval

All procedures performed in studies involving human participants were in accordance with the ethical standards of the institutional and/or national research committee and with the 1964 Helsinki declaration and its later amendments or comparable ethical standards. For this type of study, based exclusively on routinely collected data, formal consent is not required.

## Results

**Figure 1:**
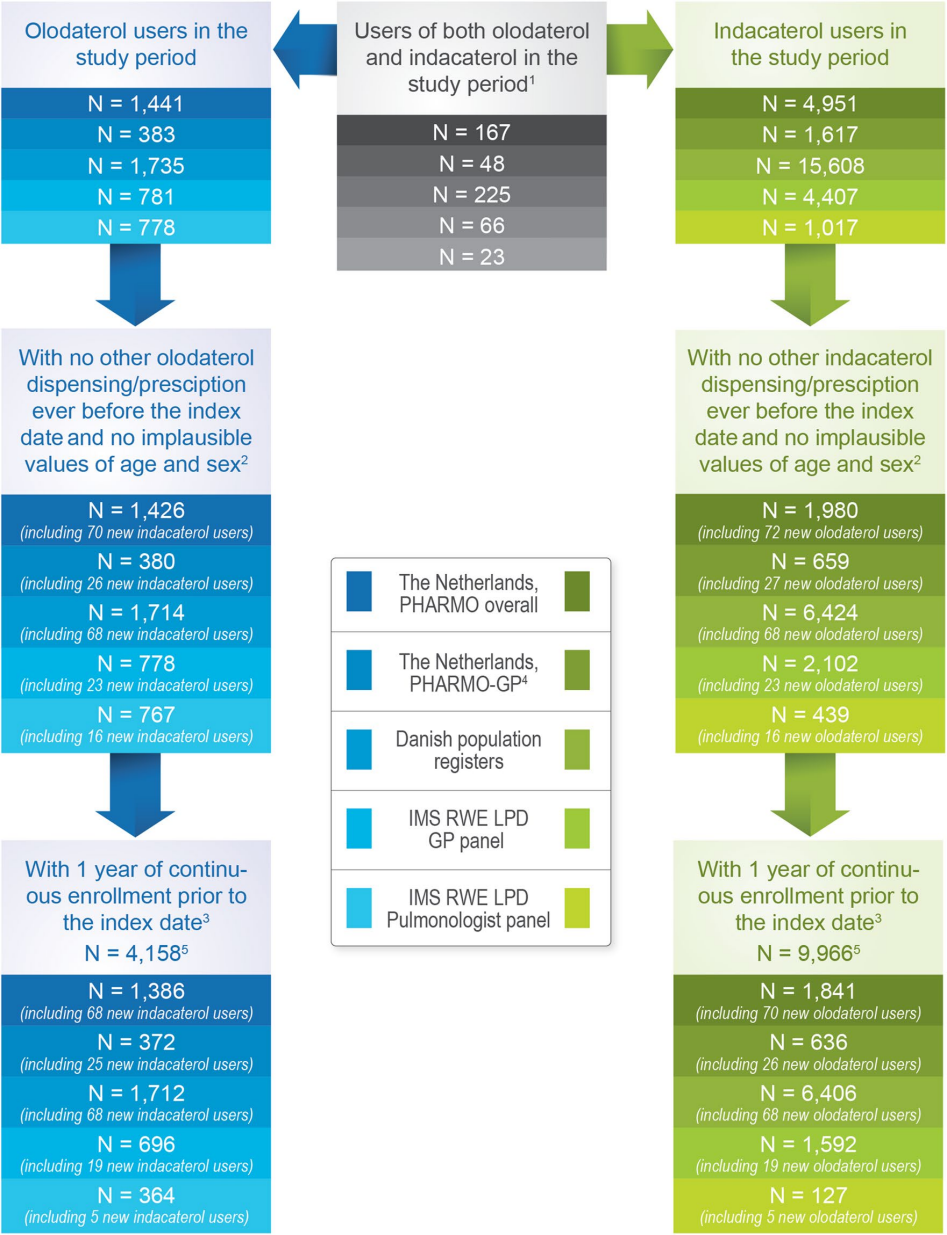
Number of users of olodaterol and indacaterol before and after fulfilling inclusion/exclusion criteria in each data source.

The study included 4,158 new users of olodaterol and 9,966 new users of indacaterol (Fig. 1) across the three countries. Among users of olodaterol or indacaterol, the main reason for exclusion was not being a new user.

### Demographic and lifestyle characteristics

The median age ranged from 63 years (IMS RWE LPD GP panel) to 71 years (Denmark) in new users of olodaterol and ranged from 63 years (IMS RWE LPD GP panel) to 69 years (Denmark) in new users of indacaterol (Table 1). Overall, the proportion of females and males among new users of olodaterol and indacaterol was close to 50% in all data sources except in the IMS RWE LPD pulmonologist panel, where 64.6% of the new users of olodaterol and 66.9% of the new users of indacaterol were males. In most data sources, a high proportion of patients had no information recorded on lifestyle characteristics. Among patients with recorded information on smoking history, the proportion of those who were current smokers was around 50% in PHARMO-GP and in the IMS RWE LPD GP panel and was higher in the IMS RWE LPD pulmonologist panel, which reported that 90.7% of the new users of olodaterol and 72.2% of the new users of indacaterol were current smokers. There was incomplete recording of obesity in all data sources. In the subset of patients in PHARMO-GP for whom information on obesity was available (72.8% of those new users of olodaterol and 64.6% among new users of indacaterol), the proportion of overweight or obese patients was 66.1% among new users of olodaterol and was 66.7% among new users of indacaterol. The proportion of patients with a recorded diagnosis of alcohol-related disorders was below 10% in all health care databases for both olodaterol and indacaterol new users.

**Table 1:** Description of demographics and lifestyle variables of new users of olodaterol and indacaterol.

	PHARMO Overall		PHARMO-GP		Danish population registers		IMS RWE LPD GP panel		IMS RWE LPD pulmonologist panel	
	Olodaterol (N = 1,386)	Indacaterol (N = 1,841)	Olodaterol (N = 372)	Indacaterol (N = 636)	Olodaterol (N = 1,712)	Indacaterol (N = 6,406)	Olodaterol (N = 696)	Indacaterol (N = 1,592)	Olodaterol (N = 364)	Indacaterol (N = 127)
Males, n (%)	684 (49.4)	954 (51.8)	175 (47.0)	334 (52.5)	735 (42.9)	3,082 (48.1)	374 (53.7)	896 (56.3)	235 (64.6)	85 (66.9)
Age, median (IQR)	68.0 (60.0–75.0)	68.0 (60.0–75.0)	68.0 (59.5–76.0)	68.0 (59.0–74.5)	71.0 (64.0–78.5)	69.0 (61.2–76.5)	63.0 (53.0–72.0)	63.0 (53.0–73.0)	67.0 (59.0–75.0)	67.0 (59.0–75.0)
**Smoking history**, **n (%)**^**a**^										
Current (or yes)	—	—	116 (31.2)	194 (30.5)	—	—	183 (26.3)	406 (25.5)	136 (37.4)	13 (10.2)
Former	—	—	82 (22.0)	108 (17.0)	—	—	—	—	—	—
Never (or no)	—	—	41 (11.0)	64 (10.1)	—	—	236 (33.9)	496 (31.2)	14 (3.8)	14 (3.8)
Unknown	—	—	133 (35.8)	270 (42.5)	—	—	277 (39.8)	690 (43.3)	214 (58.8)	214 (58.8)
**Obesity or overweight**, **n (%)**										
Yes	208 (15.0)	307 (16.7)	179 (48.1)	274 (43.1)	132 (7.7)	385 (6.0)	140 (20.2)	293 (18.4)	79 (21.7)	12 (9.4)
No	93 (6.7)	138 (7.5)	92 (24.7)	137 (21.5)	NA	NA	80 (11.5)	179 (11.2)	44 (12.1)	3 (2.4)
Unknown	1,085 (78.3)	1,396 (75.8)	101 (27.2)	225 (35.4)	1,580 (92.3)	6,021 (94.0)	476 (68.4)	1,120 (70.4)	241 (66.2)	112 (88.2)
**Alcohol-related disorders (yes),** **n (%)**^**b**^	73 (5.3)	83 (4.5)	17 (4.6)	27 (4.2)	146 (8.5)	600 (9.4)	35 (5.0)	86 (5.4)	0 (0.0)	1 (0.8)

GP = general practitioner; IMS RWE LPD = IMS Health Information Solutions Real-World Evidence Longitudinal Patient Database; IQR = interquartile range; PHARMO = PHARMO Database Network; PHARMO-GP = PHARMO General Practitioner Database.

^a^There was no information on smoking status at index date in PHARMO overall, and in the Danish Registries.

^b^Alcohol-related disorders was used as a proxy of history of heavy drinking due to limited availability of data on alcohol consumption at index date in most data sources.

### Comorbidities (respiratory-related and nonrespiratory-related) ever before the index date

In general, the proportion of patients with recorded respiratory and nonrespiratory diseases was higher among new users of olodaterol than among new users of indacaterol. Frequent respiratory comorbidities other than COPD were asthma, pneumonia, and other respiratory conditions (Table 2). The most frequent nonrespiratory diseases among new users of olodaterol and indacaterol across data sources were hypertension, ischemic heart disease, other forms of heart disease, arrhythmias, renal disease, diabetes mellitus, and malignancies (Table 2).

**Table 2:** Number and proportion of patients with medical history of respiratory and nonrespiratory diseases, at any time before index date and up to 30 days after and number and proportion of users of respiratory and nonrespiratory comedications within 12 months prior to index date among new users of olodaterol and indacaterol, by data source.

	PHARMO Overall		PHARMO-GP		Danish population registers		IMS RWE LPD GP panel		IMS RWE LPD pulmonologist panel	
	Olodaterol (N = 1,386)	Indacaterol (N = 1,841)	Olodaterol (N = 372)	Indacaterol (N = 636)	Olodaterol (N = 1,712)	Indacaterol (N = 6,406)	Olodaterol (N = 696)	Indacaterol (N = 1,592)	Olodaterol (N = 364)	Indacaterol (N = 127)
**Respiratory diseases**, **n (%)**										
COPD	663 (47.8)	653 (35.5)	276 (74.2)	407 (64.0)	1,118 (65.3)	1,840 (28.7)	374 (53.7)	847 (53.2)	283 (77.7)	89 (70.1)
Asthma	166 (12.0)	202 (11.0)	107 (28.8)	166 (26.1)	261 (15.2)	542 (8.5)	218 (31.3)	457 (28.7)	100 (27.5)	26 (20.5)
Pneumonia	210 (15.2)	184 (10.0)	52 (14.0)	87 (13.7)	565 (33.0)	1,200 (18.7)	186 (26.7)	319 (20.0)	46 (12.6)	9 (7.1)
Other respiratory conditions	191 (13.8)	186 (10.1)	43 (11.6)	60 (9.4)	445 (26.0)	649 (10.1)	60 (8.6)	123 (7.7)	102 (28.0)	19 (15.0)
**Nonrespiratory diseases**, **n (%)**										
Ischemic heart disease	213 (15.4)	258 (14.0)	49 (13.2)	71 (11.2)	405 (23.7)	1,236 (19.3)	88 (12.6)	176 (11.1)	28 (7.7)	7 (5.5)
Arrhythmias	171 (12.3)	179 (9.7)	48 (12.9)	78 (12.3)	269 (15.7)	796 (12.4)	84 (12.1)	187 (11.7)	23 (6.3)	5 (3.9)
Heart failure	87 (6.3)	88 (4.8)	29 (7.8)	37 (5.8)	183 (10.7)	384 (6.0)	20 (2.9)	46 (2.9)	5 (1.4)	0 (0.0)
Hypertension	261 (18.8)	291 (15.8)	96 (25.8)	157 (24.7)	560 (32.7)	1,737 (27.1)	305 (43.8)	682 (42.8)	85 (23.4)	17 (13.4)
Other forms of heart diseases	179 (12.9)	226 (12.3)	68 (18.3)	110 (17.3)	384 (22.4)	1,255 (19.6)	224 (32.2)	440 (27.6)	21 (5.8)	3 (2.4)
Cerebrovascular disease	73 (5.3)	113 (6.1)	25 (6.7)	41 (6.4)	226 (13.2)	727 (11.3)	45 (6.5)	94 (5.9)	12 (3.3)	1 (0.8)
Hyperlipidemia	95 (6.9)	124 (6.7)	47 (12.6)	77 (12.1)	235 (13.7)	135 (7.9)	193 (27.7)	455 (28.6)	29 (8.0)	8 (6.3)
Renal disease	166 (12.0)	187 (10.2)	42 (11.3)	74 (11.6)	385 (22.5)	193 (11.3)	135 (19.4)	296 (18.6)	9 (2.5)	2 (1.6)
Depressive disorders	27 (1.9)	25 (1.4)	23 (6.2)	23 (3.6)	771 (12.0)	303 (17.7)	185 (26.6)	427 (26.8)	26 (7.1)	4 (3.1)
Diabetes mellitus	140 (10.1)	167 (9.1)	42 (11.3)	78 (12.3)	1,061 (16.6)	543 (8.5)	109 (15.7)	236 (14.8)	35 (9.6)	10 (7.9)
Malignancy	179 (12.9)	194 (10.5)	49 (13.2)	77 (12.1)	771 (12.0)	1,047 (16.3)	85 (12.2)	179 (11.2)	44 (12.1)	6 (4.7)
**Respiratory medications**, **n (%)**										
LABA^a^	382 (27.6)	185 (10.0)	104 (28.0)	61 (9.6)	437 (25.5)	351 (5.5)	97 (13.9)	64 (4.0)	51 (14.0)	5 (3.9)
LABA/ICS	514 (37.1)	519 (28.2)	137 (36.8)	170 (26.7)	687 (40.1)	1,177 (18.4)	197 (28.3)	334 (21.0)	68 (18.7)	24 (18.9)
LABA/LAMA	81 (5.8)	5 (0.3)	21 (5.6)	2 (0.3)	190 (11.1)	58 (0.9)	59 (8.5)	2 (0.1)	46 (12.6)	1 (0.8)
LAMA	1,088 (78.5)	1,081 (58.7)	275 (73.9)	368 (57.9)	1,228 (71.7)	1,536 (24.0)	296 (42.5)	282 (17.7)	235 (64.6)	39 (30.7)
SAMA	174 (12.6)	193 (10.5)	32 (8.6)	70 (11.0)	12 (0.7)	19 (0.3)	18 (2.6)	13 (0.8)	72 (19.8)	3 (2.4)
SABA	623 (44.9)	643 (34.9)	140 (37.6)	210 (33.0)	991 (57.9)	2,482 (38.7)	176 (25.3)	349 (21.9)	119 (32.7)	19 (15.0)
SABA/SAMA	105 (7.6)	54 (2.9)	25 (6.7)	9 (1.4)	98 (5.7)	95 (1.5)	23 (3.3)	38 (2.4)	20 (5.5)	15 (11.8)
ICS	378 (27.3)	334 (18.1)	77 (20.7)	95 (14.9)	302 (17.6)	667 (10.4)	117 (16.8)	206 (12.9)	54 (14.8)	18 (14.2)
Systemic glucocorticosteroids	685 (49.4)	617 (33.5)	149 (40.1)	223 (35.1)	662 (38.7)	1,188 (18.5)	287 (41.2)	515 (32.3)	54 (14.8)	13 (10.2)
**Nonrespiratory medications**, **n (%)**										
Cardiovascular medications	900 (64.9)	1,174 (63.8)	246 (66.1)	396 (62.3)	1,245 (72.7)	4,288 (66.9)	414 (59.5)	865 (54.3)	11 (3.0)	2 (1.6)
Systemic antibacterials	824 (59.5)	928 (50.4)	197 (53.0)	317 (49.8)	1,179 (68.9)	3,493 (54.5)	467 (67.1)	919 (57.7)	84 (23.1)	18 (14.2)
Proton pump inhibitors	765 (55.2)	927 (50.4)	195 (52.4)	337 (53.0)	610 (35.6)	1,859 (29.0)	329 (47.3)	676 (42.5)	21 (5.8)	5 (3.9)
Antithrombotic agents	602 (43.4)	761 (41.3)	145 (39.0)	261 (41.0)	710 (41.5)	2,225 (34.7)	195 (28.0)	362 (22.7)	2 (0.5)	0 (0.0)
Drugs for musculoskeletal system	—	—	—	—	433 (25.3)	1,511 (23.6)	219 (31.5)	428 (26.9)	1 (0.3)	0 (0.0)

GP = general practitioner; ICS = inhaled glucocorticosteroids; IMS RWE LPD = IMS Health Information Solutions Real-World Evidence Longitudinal Patient Database; LABA = inhaled long-acting beta2-agonist; LAMA = long-acting muscarinic antagonist; NA = not available; PHARMO = PHARMO Database Network; PHARMO-GP = PHARMO General Practitioner Database; SABA = short-acting beta2-agonists; SAMA = short-acting muscarinic antagonists.

^a^Prior use of LABA includes use of indacaterol for those in the olodaterol group but not indacaterol and vice versa.

Note: percentages from column totals are displayed.

### Comedications (respiratory-related and nonrespiratory-related) prescribed/dispensed within 1 year before the index date

In general, prescriptions/dispensings for respiratory and nonrespiratory medications in the year before the index date were more frequent among new users of olodaterol than among new users of indacaterol in all data sources (Table 2). The most frequent prescription/dispensing for respiratory medications in the year before the index date among new users of olodaterol and indacaterol was LAMA, followed by systemic glucocorticosteroids, short-acting beta2-agonist (SABA), LABA/ICS, nasal glucocorticosteroids, and LABA. The most frequent prescriptions/dispensings for nonrespiratory medications among new users of olodaterol and indacaterol were, in general, cardiovascular medications, followed by systemic antibacterial, proton pump inhibitors, antithrombotic agents, and drugs for musculoskeletal system.

### COPD severity among new users with COPD at the index date

Severity of COPD among new users of olodaterol and indacaterol aged 40 years or older with COPD in PHARMO overall, PHARMO-GP, and in Denmark is presented in Table 3. The proportion of patients aged 40 years or older with severe or very severe COPD was lowest in PHARMO-GP and highest in Denmark, ranging from 41.7% (PHARMO-GP) to 67.1% (Denmark) among new users of olodaterol and from 42.5% (PHARMO-GP) to 53.0% (PHARMO overall) among new users of indacaterol.

**Table 3:** COPD severity among new users aged 40 years or older with COPD at the index date, by study medication and by data source.

COPD severity categories, N (%)^a^	PHARMO Overall		PHARMO-GP		Danish population registers	
	Olodaterol (N = 1,386)	Indacaterol (N = 1,841)	Olodaterol (N = 372)	Indacaterol (N = 636)	Olodaterol (N = 1,712)	Indacaterol (N = 6,406)
**N total**	662	648	276	405	1,115	1,835
**Mild**^b^	23 (3.5)	63 (9.7)	17 (6.2)	53 (13.1)	67 (6.0)	390 (21.3)
**Moderate**^c^	255 (38.5)	242 (37.3)	144 (52.2)	180 (44.4)	300 (26.9)	503 (27.4)
**Severe**^d^	264 (39.9)	226 (34.9)	69 (25.0)	94 (23.2)	572 (51.3)	779 (42.5)
At least one hospitalization for COPD exacerbation in prior year	192 (72.7)	177 (78.3)	35 (50.7)	55 (58.5)	356 (62.2)	450 (57.8)
At least two COPD exacerbations without hospitalization, where COPD exacerbation is defined by any of the following:	98 (37.1)	47 (20.8)	28 (40.6)	20 (21.3)	392 (68.5)	490 (62.9)
A diagnosis of COPD exacerbation without hospitalization	11 (11.2)	6 (12.8)	11 (39.3)	6 (30.0)	32 (8.2)	40 (8.2)
A course of antibiotics for respiratory tract infections	75 (76.5)	30 (63.8)	18 (64.3)	10 (50.0)	353 (90.1)	453 (92.4)
A course of systemic glucocorticosteroids for COPD exacerbation	84 (85.7)	37 (78.7)	21 (75.0)	14 (70.0)	291 (74.2)	323 (65.9)
**Very severe**^e^	120 (18.1)	117 (18.1)	46 (16.7)	78 (19.3)	176 (15.8)	163 (8.9)
Dispensed oxygen therapy in prior year	NA	NA	NA	NA	32 (18.2)	17 (10.4)
Dispensed nebulizer therapy in prior year	38 (31.7)	14 (12.0)	6 (13.0)	2 (2.6)	45 (25.6)	16 (9.8)
Diagnosis of emphysema at any time before index date	83 (69.2)	101 (86.3)	39 (84.8)	73 (93.6)	116 (65.9)	135 (82.8)

COPD = chronic obstructive pulmonary disease; LABA = inhaled long-acting beta2-agonist; NA = not available; PHARMO = PHARMO Database Network; PHARMO-GP = PHARMO General Practitioner Database.

^a^Severity categories were mutually exclusive and patients that fulfill criteria for more than one category were classified as being in the most severe category. The proportion of patients in each COPD severity category was calculated over the total number of new users aged 40 years or older with COPD at the index date. For indented subcategories of the COPD severity groups, the proportion of patients was calculated over the number of patients in the non-indented category or subcategory.

^b^Patients were classified in this category when they did not fulfill criteria for very severe, severe, or moderate.

^c^At least two prescriptions/dispensings of the same COPD drug class with a maximum interval of 6 months in the 12 months before index date.

^d^Patients with at least one of the listed criteria for severe in the prior year.

^e^Patients with at least one of the listed criteria for very severe.

### Frequency of off-label use of olodaterol and indacaterol

**Figure 2:**
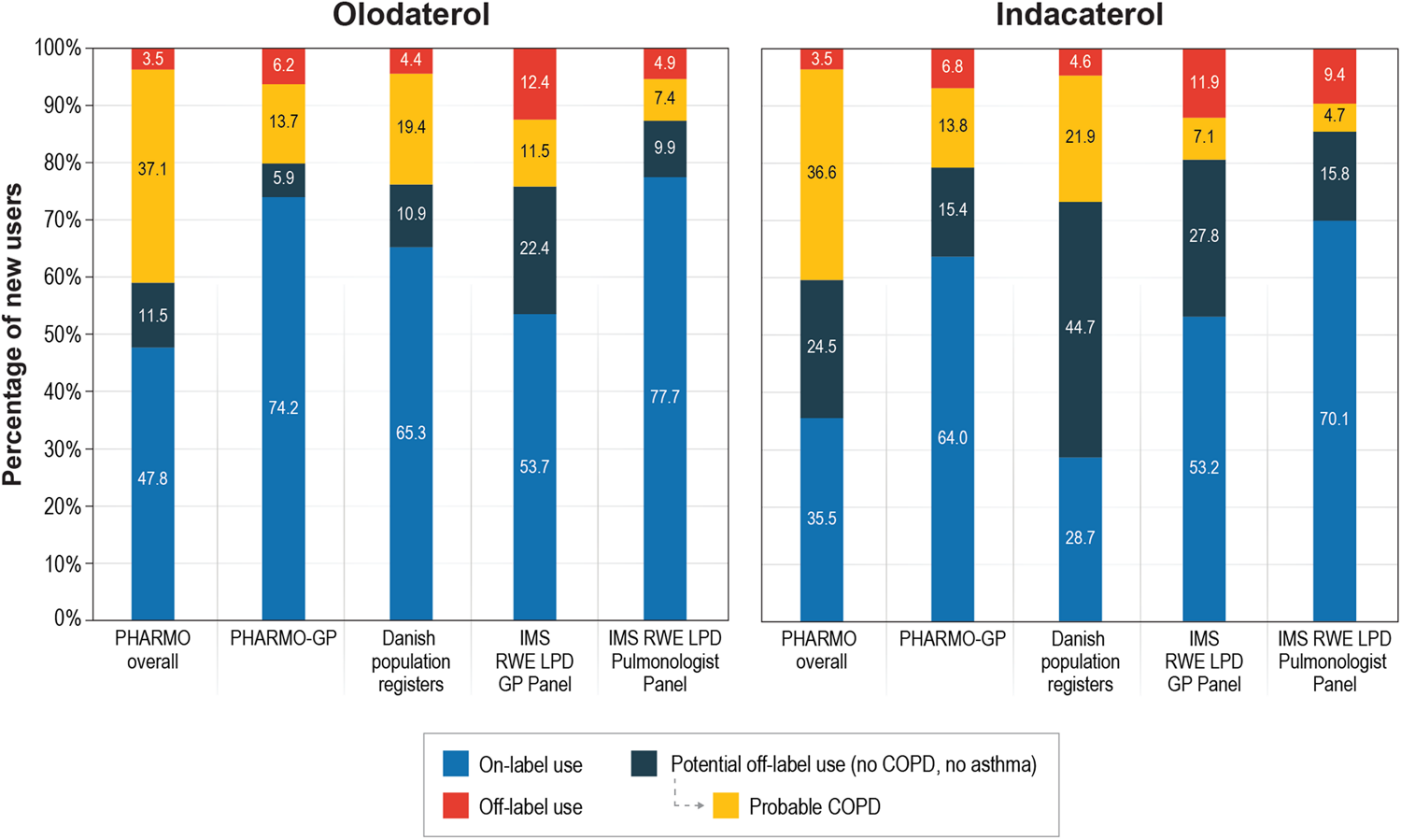
Frequency of off-label, on-label and potential off-label use in new users of olodaterol and indacaterol.

The proportion of patients with off-label prescription/dispensing ranged from 3.5% (PHARMO overall) to 12.4% (IMS RWE LPD GP panel) in new users of olodaterol and ranged from 3.5% (PHARMO overall) to 11.9% (IMS RWE LPD GP panel) in new users of indacaterol (Fig. 2 and Supplemental Tables 5 and 6). On-label prescribing/dispensing ranged from 47.8% (PHARMO overall) to 77.7% (IMS RWE LPD pulmonologist panel) in new users of olodaterol and from 28.7% (Denmark) to 70.1% (IMS RWE LPD pulmonologist panel) in new users of indacaterol. The proportion of new users classified as potential off-label prescription/dispensing ranged from 17.3% in the IMS RWE LPD pulmonologist panel to 48.6% in PHARMO overall for olodaterol and 20.5% in the IMS RWE LPD pulmonologist panel to 66.6% in Denmark for indacaterol. Among those patients classified as having potential off-label prescription/dispensing, between 33.9% (IMS RWE LPD GP panel) and 76.3% (PHARMO overall) of the new users of olodaterol and between 20.3% (IMS RWE LPD GP panel) and 60.0% (PHARMO overall) of the new users of indacaterol were classified as having probable COPD.

## Conclusion

The prevalence of off-label use of olodaterol (3.5% to 6.2%) and indacaterol (3.5% to 9.4%) reported in the present study was at the lower bound of the range of off-label use reported by the European Commission (EC) in 2017. In this review, off-label use of different medications, either specific active substances or drug classes, in 13 European studies in a variety of therapeutic areas ranged from 6% to 72%. Off-label use for respiratory medications indicated either for asthma or COPD was 69% among patients aged 18–70 years and 17% among patients aged >70 years^20^. In this study, the prevalence of off-label use of olodaterol and indacaterol was only somewhat higher in the IMS RWE LPD GP panel (12.4% for olodaterol and 11.9% for indacaterol). No notable differences in off-label use were observed between olodaterol and indacaterol.

Results of the present study indicate that new users of olodaterol and indacaterol represent an elderly population, with a similar sex distribution and a high prevalence of respiratory and nonrespiratory comorbidities and use of comedications. In general, age and sex distribution of new users of olodaterol were similar to that of new users of indacaterol, and were in line with findings in former studies^19,21–23^. A high degree of data on lifestyle characteristics were missing due to type and availability of information in the health care databases. New users of olodaterol had more comorbidities and used more comedications than new users of indacaterol. In general, the frequency of comorbidities among new users of olodaterol and indacaterol was in the range of that reported in the literature for patients with COPD, although the prevalence of most nonrespiratory comorbidities such as hypertension or ischemic heart disease was lower than expected in the IMS RWE LPD panels^19,22–24^. Differences in the type and availability of data in each data source and differences in the duration of the look-back period may explain part of the differences observed between data sources. The results indicate that olodaterol, the newer medication, tends to be prescribed to patients who are less stable and need medication changes or to patients with more severe COPD as compared with older medications with similar indications of use (i.e. indacaterol). Differences in the distribution of COPD severity by data source can also be explained by the type and availability of data, i.e., in Denmark only patients who have had a hospital contact for COPD could have a recorded diagnosis of COPD, thus diagnosis of COPD recorded among those patients managed in primary care could not be captured. The proportion of use of other respiratory medications in PHARMO overall, PHARMO-GP, and Denmark was similar to proportions of these medications described in the literature, except that of prior use of ICS, which was lower^19,21^.

The study included a high number of users across different health care systems and data sources in Europe. Performing the study across several data sources increased the number of new users being evaluated and allowed assessment of potential differences in the patterns of use between countries for the medications of interest. Heterogeneity in the type and completeness of the information in these data sources may be driving, at least in part, the differences observed between them. However, the results must be evaluated in the context of the study’s two main limitations. First, the data sources used in this study provided detailed information on prescribed/dispensed medications but not on the actual use of the medications, and this may lead to exposure misclassification, which can be worst in patients with COPD, a disease with frequently low adherence and persistence to treatment^25,26^. This type of misclassification is more likely to occur in the IMS RWE LPD panels that have information on prescriptions rather than in the PHARMO and Danish data sources that have information on dispensings^27^. Second, the recorded COPD and asthma diagnosis might be incomplete. In the present study, on-label use was identified only by recorded diagnoses for COPD. It is likely that this method resulted in an underestimation of the prevalence of on-label use due to the potential incomplete recording of COPD diagnosis in the health care data sources [18]. A high proportion of new users of olodaterol and indacaterol had neither recorded COPD nor asthma diagnosis codes and were classified as “potential off-label use”. To untangle the potential misclassification due to incomplete recording, a medication algorithm was used to identify patients that were likely to have COPD. A high proportion of up to 76.3% of the patients in PHARMO overall without a recorded diagnosis of COPD or asthma had “probable COPD” based on the medication proxy, suggesting that these patients are likely using the drug on-label versus what had been identified in the participating health care databases. Results also suggest that recording of COPD is more incomplete in the IMS RWE LPD GP panel, in the PHARMO overall, and in Denmark compared with PHARMO-GP and the IMS RWE LPD pulmonologist panel. Incomplete recording of COPD diagnosis in the IMS RWE LPD GP panel is likely to occur because of the nature of the database, where only day-to-day diagnoses are recorded and there is no incentive to record all of a patient’s comorbidities. In PHARMO overall, only 25% have primary care data, and in Denmark there is no access to primary care data. The diagnosis and treatment of COPD is often managed by GPs outside hospitals, implying that hospital-based diagnoses capture primarily patients with more severe COPD. Therefore, incomplete recording of COPD is expected when only hospital data and not primary care data is available. This issue is supported by literature showing that COPD prevalence in Denmark was estimated to approximately 4.3% in hospital settings, while it was estimated to be 12% in primary care settings^8,28^. Even in health care data sources with access to primary care data, recording may be incomplete when recording is not mandatory for the GPs. Incomplete recording is also supported by the fact that the proportion of patients classified as having “probable COPD” is high in all data sources, but higher in data sources with no primary care data.

Use of olodaterol and indacaterol among patients with asthma only (and no COPD) is expected to be limited to patients who have failed to experience symptom improvement with other LABAs, who may find presentation dosage or device to be less convenient, or who have had drug substance-specific adverse events or contraindications. Routinely collected data used in this study do not specifically record indication for use or patient preference; therefore, the reported level of off-label use is at least partially attributable to the limitations of the data sources. Similarly, we cannot completely rule out potential misclassification of COPD as asthma, although it is less likely that true asthma was misclassified as COPD.

Overall, this study provides no evidence of a major concern for an off-label use of olodaterol or indacaterol in asthma or pediatric use of olodaterol or indacaterol in Europe. The lack of direct evidence of prescribed indication (potential off-label use) for a high proportion of patients is a limitation for some data sources, although indirect evidence supports that off-label use is low. Finally, given that asthma management guidelines have consistently and strongly recommended that LABAs should be used only in combination with ICS^13^, it is expected that use of olodaterol and indacaterol among patients with asthma only is done with concomitant use of ICS, although this was not evaluated in our study.

## Supplementary information

SUPPLEMENTARY APPENDIX.

## Data Availability

This study uses the national health care databases, panels of physicians, and registries. Only the aggregated data, in the form of study results in the main manuscript and in the supplementary material can be shared.
