# Supplementary material for: No Evidence of Off-label Use of Olodaterol and Indacaterol in Denmark, France, and the Netherlands: A Drug Utilization Study
Source: Sci Rep. 2020 Jan 17;10:586. doi: 10.1038/s41598-019-57397-5 (PMC6968970; doi:10.1038/s41598-019-57397-5)
Supplement: Supplementary file 1 — SUPPLEMENTARY APPENDIX. [file 41598_2019_57397_MOESM1_ESM.docx]

**SUPPLEMENTARY APPENDIX**

**Title:** No Evidence of Off-label Use of Olodaterol and Indacaterol in Denmark, France, and the Netherlands: A Drug Utilization Study

**Author Names:**

Cristina Rebordosa, MD, PhD^1^; Eline Houben, MSc^2^; Kristina Laugesen, MD^3^; Ulrich Bothner, MD, MSc^5^, Jukka Montonen, PhD^5^; Jaume Aguado, PhD^1^; Jetty A. Overbeek, MSc^2^; Vera Ehrenstein, MPH, DSc^3^; Joelle Asmar, PhD^4^; Laura Wallace MPH^6^; and Alicia W. Gilsenan, PhD^1^

**Author Affiliations:**

^1^RTI Health Solutions, Barcelona, Spain and Research Triangle Park, NC

^2^PHARMO Institute for Drug Outcomes Research, Utrecht, Netherlands

^3^Department of Clinical Epidemiology, Aarhus University Hospital, Aarhus, Denmark

^4^IQVIA, RWI, Paris, France

^5^Boehringer Ingelheim International GmbH, Ingelheim am Rhein, Germany

^6^ Boehringer Ingelheim International GmbH, Ridgefield CT USA

**Author for Correspondence:**

Cristina Rebordosa, MD, PhD

RTI Health Solutions, Av. Diagonal 605, 9-1 08028 Barcelona, Spain

Telephone: + 3493.362.2807

Fax: + 3493.414.2610

E‑mail: [crebordosa@rti.org](mailto:crebordosa@rti.org)

Supplemental Table 1. Selected characteristics of the study databases

| Type of data | PHARMO,  the Netherlands | Danish population registers, Denmark | IMS RWE LPD, France |
| --- | --- | --- | --- |
| Hospital inpatient discharge diagnoses | Yes | Yes | No |
| Hospital inpatient procedures | Yes | Yes | No |
| Hospital/clinic outpatient diagnoses | No | Yes | No |
| General practitioner outpatient diagnoses | Yes, in data subset PHARMO‑GP^a^ | No | Yes (a panel of 1,200 general practitioners)^b^ |
| Specialist diagnoses | Yes, those in the hospital (no outpatient hospital or nonhospital clinics) or as communicated to and recorded by the GP in PHARMO‑GP^a^ | Yes, those in the hospital (inpatient diagnoses) and hospital outpatient clinics (outpatient diagnoses); not outside the hospital | Yes (a panel of 40 pulmonologists)^c^ |
| Type of codes for diagnoses | Hospital: ICD-9-CM and ICD-10^d^  Primary health care: ICPC^e^  Examination (“WCIA”) codes^f^ | ICD-10 | Local in-house thesaurus that can be mapped to ICD-10 |
| Pharmacy-dispensed medications | Yes | Yes | No |
| Prescribed medications | Yes, in data subset PHARMO‑GP^a^ | No | Yes |
| Type of codes for medications | ATC | ATC | Claude Bernard/ EphMRA mapped to ATC |

ATC = Anatomical Therapeutic Chemical; EphMRA = European Pharmaceutical Market Research Association; GP = general practitioner; ICD-9-CM = *International Classification of Diseases, 9th Revision, Clinical Modification*; ICD‑10 = *International Statistical Classification of Diseases and Related Health Problems, 10th Revision*; ICPC = International Classification of Primary Care; PHARMO = PHARMO Database Network; PHARMO‑GP = PHARMO General Practitioner Database; IMS RWE LPD = IMS Health Information Solutions Real-World Evidence Longitudinal Patient Database; WCIA = Werkgroep Coordinatie Informatisering en Automatisering.

^a^ For this study, data from the Out-patient Pharmacy Database and the Hospitalisation Database are available for the overall source population (approximately 4 million patients covered); the subcohort PHARMO‑GP includes approximately 1 million patients with data in the Out-patient Pharmacy Database, the Hospitalisation Database, and the General Practitioner (GP) Database. The Out-patient Pharmacy Database was used to identify medication use.

^b^ The GP panel covers a population of approximately 1.8 million active patients.

^c^ The pulmonologist panel covers a population of approximately 55,000 active patients.

^d^ Hospital codes in PHARMO before 2014 were ICD-9-CM. PHARMO will work with ICD-9-CM codes and map them to ICD-10 codes before conducting the analysis.

^e^ Available for PHARMO-GP, a data subset of PHARMO with information from GPs.

^f^ Examination (“WCIA”) codes are recorded in the General Practitioner (GP) Database, which comprises data from electronic patient records registered by GPs. These records include information on GP examinations (i.e., assessments done by the GP, regarding diagnoses and symptoms, laboratory or function tests), which are coded according to the Dutch national standard coding system (WCIA) of the Dutch National Association of General Practitioners and The Dutch College of General Practitioners. This coding system is available also on http://aut.nhg.org/labcodeviewer/ (only available in Dutch).

Supplemental Table 2. ICD‑10 and ICPC diagnosis codes to identify patients with COPD and asthma

| ICD‑10 code description | ICD‑10 code^a^ | ICPC code^a^ |
| --- | --- | --- |
| COPD | J41-J44 | R95 |
| Chronic bronchitis^b^ | J41-J42 | R78, R91 |
| Emphysema | J43 | — |
| Other COPD | J44 | — |
| Asthma | J45, J46 | R96 |

COPD = chronic obstructive pulmonary disease; ICD‑10 = *International Statistical Classification of Diseases and Related Health Problems, 10th Revision*; ICPC = International Classification of Primary Care.

^a^ Data sources use different coding systems with different degrees of granularity; thus, subclassifications will be adapted to each database.

^b^ The use of ICD-10 code J40 “Bronchitis, not specified as acute or chronic” to define COPD will be assessed in the data source–specific definitions.

Supplemental Table 3. Demographic and lifestyle variables: proposed definition, units, and categories by data source

| Variable | PHARMO,  the Netherlands | Danish population registers, Denmark | IMS RWE LPD, France |
| --- | --- | --- | --- |
| Smoking status | Smoking status (current, former, never, and missing) in PHARMO‑GP | NA | Smoker/nonsmoker |
| Alcohol consumption | Alcohol-related disorder (yes/no) in PHARMO‑GP | Alcohol-related disorder (yes/no) | Alcohol-related disorder (yes/no) |
| Body mass index (kg/m^2^), overweight/obesity | BMI and diagnosis of obesity or overweight in PHARMO‑GP | Inpatient or outpatient hospital diagnosis of obesity or overweight | BMI and categories of BMI |

BMI = body mass index; IMS RWE LPD = IMS Health Information Solutions Real-World Evidence Longitudinal Patient Database; NA = not available; PHARMO = PHARMO Database Network; PHARMO‑GP = PHARMO General Practitioner Database.

Supplemental Table 4. Definition criteria of COPD severity (adapted primarily from Verhamme, et al. ^1^)

| Severity of COPD | Definition |
| --- | --- |
| Mild | Less than two prescriptions/dispensings of the same COPD drug class^a^ with a maximum interval of 6 months in the 12 months before the index date |
| Moderate | Regular bronchodilator treatment, defined as having at least two prescriptions/dispensings of the same COPD drug class with a maximum interval of 6 months in the 12 months before the index date^b,c^ |
| Severe | Occurrence of at least one hospitalization for COPD exacerbation in the year before the index date,  OR  Occurrence of at least two COPD exacerbations without hospitalization in the year before the index date, where each COPD exacerbation was defined by *any* of the following^d,e^:   - A course of antibiotics for respiratory tract infections^c^ - A course of systemic glucocorticosteroids for the treatment of COPD exacerbation^c^ - A diagnosis of COPD exacerbation without hospitalization |
| Very severe | Occurrence of at least one of the following events in the year before the index date, unless other time period is specified:   - Dispensed oxygen therapy^b,c^ - Dispensed nebulizer therapy^b,c^ - Diagnosis of emphysema at any time before the index date^c^ |

COPD = chronic obstructive pulmonary disease.

Sources: modified from Verhamme et al., 2012 ^1^; Soriano et al., 2001 ^2^; Curkendall et al., 2006 ^3^; and GOLD, 2016 ^4^.

^a^ The following bronchodilator classes were considered: (1) bronchodilators—inhaled short-acting muscarinic antagonists (SAMAs), inhaled long-acting muscarinic antagonists (LAMAs), inhaled short-acting beta2-agonists (SABAs), inhaled LABAs, and fixed combinations of SABA and SAMA; (2) inhaled glucocorticosteroids (ICSs)—ICS alone, fixed combinations of SABA and ICS, and fixed combinations of LABA and ICS; (3) systemic glucocorticosteroids; (4) systemic beta2-agonists; (5) xanthines; and (6) roflumilast.

^b^ Severity criteria also included in definition from Soriano et al., 2001 ^2^.

^c^ Severity criteria also included in definition from Curkendall et al., 2006 ^3^.

^d^ Severity criteria modified to align with the Global Initiative for Chronic Obstructive Lung Disease (GOLD) 2016 definition for severity categories C and D ^4^. If two or more of these criteria occurred within a 21-day time period, they were considered part of the same exacerbation episode and counted only once. If one or more of these criteria occurred within a 21-day period of a hospitalization for COPD, this was considered part of the same “COPD exacerbation with hospitalization” episode.

^e^ A course of antibiotics or systemic glucocorticosteroids was defined as one involving consecutive prescriptions/dispensings with less than 7 days between the end of days of supply of one prescription/dispensing and the date of the next prescription/dispensing.

Supplemental Table 5. Prevalence of on- and off-label use for olodaterol new users of all ages

| On-label, off-label, and potential off-label categories by study medication | PHARMO overall | PHARMO-GP | Danish population registers, Denmark | IMS RWE LPD GP Panel | IMS RWE LPD Pulmonologist Panel |
| --- | --- | --- | --- | --- | --- |
|  | n (%^a^) | n (%^a^) | n (%^a^) | n (%^a^) | n (%^a^) |
| Olodaterol new users | 1,386 | 372 | 1,712 | 696 | 364 |
| **On-label (patients aged 18 years or older with a recorded COPD diagnosis code)** | **663 (47.8)** | **276 (74.2)** | **1,118 (65.3)** | **374 (53.7)** | **283 (77.7)** |
| COPD (no asthma) | 545 (39.3) | 192 (51.6) | 932 (54.4) | 242 (34.8) | 201 (55.2) |
| COPD and asthma | 118 (8.5) | 84 (22.6) | 186 (10.9) | 132 (19.0) | 82 (22.5) |
| **Potential off-label (patients aged 18 years or older with no recorded COPD diagnosis code and no asthma diagnosis code)^b^** | **674 (48.6)** | **73 (19.6)** | **519 (30.3)** | **236 (33.9)** | **63 (17.3)** |
| “Probable COPD”^c^ | 514 (37.1) | 51 (13.7) | 332 (19.4) | 80 (11.5) | 27 (7.4) |
| Pneumonia | 38 (2.7) | 7 (1.9) | 81 (4.7) | 17 (2.4) | 2 (0.5) |
| Allergic rhinitis | 2 (0.1) | 2 (0.5) | 5 (0.3) | 10 (1.4) | 1 (0.3) |
| Acute bronchitis or bronchiolitis | 5 (0.4) | 3 (0.8) | 10 (0.6) | 4 (0.6) | 10 (2.7) |
| Bronchiectasis | 2 (0.1) | 0 (0.0) | 10 (0.6) | 2 (0.3) | 0 (0.0) |
| Lung diseases due to external agents | 0 (0.0) | 0 (0.0) | < 5 | 12 (1.7) | 10 (2.7) |
| Other respiratory conditions | 43 (3.1) | 5 (1.3) | 63 (3.7) | 84 (12.1) | 4 (1.1) |
| No history of recorded respiratory conditions and no “probable COPD” | 138 (10.0) | 17 (4.6) | 130 (7.6) | 102 (14.7) | 26 (7.1) |
| **Off-label (patients aged 17 years or younger or patients aged 18 years or older with no recorded COPD diagnosis but with a diagnosis of asthma)** | **49 (3.5)** | **23 (6.2)** | **75 (4.4)** | **86 (12.4)** | **18 (4.9)** |
| Patients aged 17 years or younger | 1 (< 0.1) | 0 (0.0) | < 5 | 0 (0.0) | 0 (0.0) |
| Asthma only (no COPD) | 48 (3.5) | 23 (6.2) | 71-74 (4.1-4.4) | 86 (12.4) | 18 (4.9) |

COPD = chronic obstructive pulmonary disease; GP = general practitioner; ICS = inhaled glucorticosteroids; IMS RWE LPD = IMS Health Information Solutions Real‑World Evidence Longitudinal Patient Database; LABA = inhaled long-acting beta2-agonist; LAMA = long-acting muscarinic antagonist; NA = not available; PHARMO = PHARMO Database Network; PHARMO-GP = PHARMO General Practitioner Database.

Note: There were fewer than 5 patients aged 17 years or younger in the olodaterol group in the PHARMO overall and no patients aged 17 years or younger in the olodaterol group in the other data sources.

^a^ Prevalence is reported as percentage. Percentages of on-label, off-label, and potential off-label use of olodaterol, overall and in each subcategory, were calculated over the total number of olodaterol users.

^b^ Patients were classified hierarchically: first, all patients aged 17 years or younger were classified as “off-label”; second, all adult patients with a diagnosis of COPD were considered “on‑label” irrespective of whether they had a diagnosis of asthma or any other respiratory condition; third, all adults without a COPD diagnosis and with a diagnosis of asthma were classified under the group “asthma only,” irrespective of whether they had any other respiratory condition; and fourth, all adults with no COPD and no asthma diagnosis were classified as “potential off-label.” The three categories were mutually exclusive; the patients allocated in the potential off-label group could have one or more of the respiratory conditions listed or no respiratory conditions and classified as “no history of recorded respiratory conditions.” Patients with COPD were identified through diagnosis of chronic bronchitis, emphysema, or “other COPD” (i.e., COPD without specifying phenotype, e.g., ICD-10 code J44 [other chronic obstructive pulmonary disease]).

^c^ Probable COPD: patients with no recorded COPD or asthma but who had at least two prescriptions for LABA, LAMA, or ICS (or combination) after age 40 years, and none before.

Supplemental Table 6. Prevalence of on- and off-label use for indacaterol new users of all ages

| On-label, off-label, and potential off-label categories by study medication | PHARMO overall | PHARMO-GP | Danish population registers, Denmark | IMS RWE LPD GP Panel | IMS RWE LPD Pulmonologist Panel |
| --- | --- | --- | --- | --- | --- |
|  | n (%^a^) | n (%^a^) | n (%^a^) | n (%^a^) | n (%^a^) |
| Indacaterol new users | 1,841 | 636 | 6,406 | 1592 | 127 |
| **On-label (patients aged 18 years or older with a recorded COPD diagnosis code)** | **653 (35.5)** | **407 (64.0)** | **1,840 (28.7)** | **847 (53.2)** | **89 (70.1)** |
| COPD | 514 (27.9) | 284 (44.7) | 1,591 (24.8) | 579 (36.4) | 75 (59.1) |
| COPD and asthma | 139 (7.6) | 123 (19.3) | 249 (3.9) | 268 (16.8) | 14 (11.0) |
| **Potential off-label (patients aged 18 years or older with no COPD diagnosis code and no asthma diagnosis code)^b^** | **1,124 (61.1)** | **186 (29.2)** | **4,269 (66.6)** | **556 (34.9)** | **26 (20.5)** |
| “Probable COPD”^c^ | 674 (36.6) | 88 (13.8) | 1,403 (21.9) | 113 (7.1) | 6 (4.7) |
| Pneumonia | 47 (2.6) | 12 (1.9) | 499 (7.8) | 57 (3.6) | 2 (1.6) |
| Allergic rhinitis | 8 (0.4) | 8 (1.3) | 24 (0.4) | 41 (2.6) | 0 (0.0) |
| Acute bronchitis or bronchiolitis | 13 (0.7) | 11 (1.7) | 78 (1.2) | 10 (0.6) | 1 (0.8) |
| Bronchiectasis | 3 (0.2) | 0 (0.0) | 22 (0.3) | 3 (0.2) | 0 (0.0) |
| Lung diseases due to external agents | 2 (0.1) | 0 (0.0) | 11 (0.2) | 35 (2.2) | 4 (3.1) |
| Other respiratory conditions | 64 (3.5) | 12 (1.9) | 248 (3.9) | 253 (15.9) | 2 (1.6) |
| No history of recorded respiratory conditions and no “probable COPD” | 400 (21.7) | 76 (11.9) | 2,368 (37.0) | 194 (12.2) | 13 (10.2) |
| **Off-label (patients aged 17 years or younger or patients aged 18 years or older with no recorded COPD diagnosis but with a diagnosis of asthma)** | **64 (3.5)** | **43 (6.8)** | **297 (4.6)** | **189 (11.9)** | **12 (9.4)** |
| Patients aged 17 years or younger | 1 (< 0.1) | 0 (0.0) | < 5 | 1 (< 0.1) | 0 (0.0) |
| Asthma only (no COPD) | 63 (3.4) | 43 (6.8) | 293-296 (4.6-4.6) | 188 (11.8) | 12 (9.4) |

COPD = chronic obstructive pulmonary disease; GP = general practitioner; ICS = inhaled glucorticosteroids; IMS RWE LPD = IMS Health Information Solutions Real‑World Evidence Longitudinal Patient Database; LABA = inhaled long-acting beta2-agonist; LAMA = long-acting muscarinic antagonist; NA = not available; PHARMO = PHARMO Database Network; PHARMO-GP = PHARMO General Practitioner Database.

Note: There were fewer than 5 patients aged 17 years or younger in the olodaterol group in the PHARMO overall and no patients aged 17 years or younger in the olodaterol group in the other data sources.

^a^ Prevalence is reported as percentage. Percentages of on-label, off-label, and potential off-label use of olodaterol, overall and in each subcategory, were calculated over the total number of olodaterol users.

^b^ Patients were classified hierarchically: first, all patients aged 17 years or younger were classified as “off-label”; second, all adult patients with a diagnosis of COPD were considered “on‑label” irrespective of whether they had a diagnosis of asthma or any other respiratory condition; third, all adults without a COPD diagnosis and with a diagnosis of asthma were classified under the group “asthma only,” irrespective of whether they had any other respiratory condition; and fourth, all adults with no COPD and no asthma diagnosis were classified as “potential off-label.” The three categories were mutually exclusive; the patients allocated in the potential off-label group could have one or more of the respiratory conditions listed or no respiratory conditions and classified as “no history of recorded respiratory conditions.” Patients with COPD were identified through diagnosis of chronic bronchitis, emphysema, or “other COPD” (i.e., COPD without specifying phenotype, e.g., ICD-10 code J44 [other chronic obstructive pulmonary disease]).

^c^ Probable COPD: patients with no recorded COPD or asthma but who had at least two prescriptions for LABA, LAMA, or ICS (or combination) after age 40 years, and none before.

# References

1 Verhamme, K. M. *et al.* Tiotropium Handihaler and the risk of cardio- or cerebrovascular events and mortality in patients with COPD. *Pulm Pharmacol Ther* **25**, 19-26, doi:10.1016/j.pupt.2011.10.004 (2012).

2 Soriano, J. B., Maier, W. C., Visick, G. & Pride, N. B. Validation of general practitioner-diagnosed COPD in the UK General Practice Research Database. *Eur J Epidemiol* **17**, 1075-1080 (2001).

3 Curkendall, S. M. *et al.* Chronic obstructive pulmonary disease severity and cardiovascular outcomes. *Eur J Epidemiol* **21**, 803-813, doi:10.1007/s10654-006-9066-1 (2006).

4 GOLD. Global strategy for the diagnosis, management, and prevention of chronic obstructive pulmonary disease. (Global Initiative for Chronic Obstructive Lung Disease, 2016).
